# Long Non-coding RNAs Genes Polymorphisms and Their Expression Levels in Patients With Rheumatoid Arthritis

**DOI:** 10.3389/fimmu.2019.02529

**Published:** 2019-10-31

**Authors:** Tian-Ping Zhang, Bang-Qiang Zhu, Sha-Sha Tao, Yin-Guang Fan, Xiao-Mei Li, Hai-Feng Pan, Dong-Qing Ye

**Affiliations:** ^1^Anhui Province Key Laboratory of Major Autoimmune Diseases, Department of Epidemiology and Biostatistics, School of Public Health, Anhui Medical University, Hefei, China; ^2^Department of Infectious Diseases, The First Affiliated Hospital of Anhui Medical University, Hefei, China; ^3^Department of Rheumatology, The First Affiliated Hospital of University of Science and Technology of China, Hefei, China

**Keywords:** ANRIL, lnc-DC, MALAT1, ZFAS1, single nucleotide polymorphisms, rheumatoid arthritis

## Abstract

Long non-coding RNAs (lncRNAs) are increasingly recognized to play important roles in multiple autoimmune diseases. This study aimed to evaluate the association of four lncRNAs (*ANRIL, lnc-DC, MALAT1, ZFAS1*) genes single nucleotide polymorphisms (SNPs) with susceptibility to rheumatoid arthritis (RA) patients, as well as their expression levels. Seventeen SNPs of the four lncRNAs were genotyped in a cohort of 660 RA patients and 710 controls using improved multiple ligase detection reaction (iMLDR). The lncRNAs expressions in peripheral blood mononuclear cells (PBMCs) from 120 RA patients and 120 controls were detected by qRT-PCR. No significant differences were found for the allele and genotype frequencies distribution of *ANRIL* SNPs (rs1412830, rs944796, rs61271866, rs2518723, rs3217992)*, lnc-DC* SNPs (rs7217280, rs10515177)*, MALAT1* SNPs (rs619586, rs4102217, rs591291, rs11227209, rs35138901), *ZFAS1* SNPs (rs237742, rs73116127, rs6125607, rs6125608) between RA patients and normal controls (all *P* > 0.05). The genotype effects of dominant and recessive models were also evaluated, but no significant association was found. In addition, our results demonstrated that the rs944796 G allele, rs2518723 T allele, rs3217992 T allele frequencies were significantly associated with anti-CCP in RA patients (all *P* < 0.05). The haplotype CGTA frequency for *ZFAS1* was significantly higher in RA patients (*P* = 0.036). Compared with normal controls, the expression levels of ANRIL, lnc-DC, MALAT1, ZFAS1 in PBMCs were significantly reduced in RA patients (all *P* < 0.001). Moreover, ZFAS1 expression was negatively associated with CRP in RA patients (*P* = 0.002). In summary, *ANRIL, lnc-DC, MALAT1*, and *ZFAS1* genes SNPs were not associated with RA susceptibility, while altered ANRIL, lnc-DC, MALAT1, ZFAS1 levels in RA patients suggested that these lncRNAs might play a role in RA.

## Introduction

Rheumatoid arthritis (RA) is known as a common autoimmune, inflammation disease characterized by systemic manifestations of immune and inflammatory response including marginal bone erosion, inflammatory joint fluid, synovitis, and destruction of articular cartilage (1, 2). Several researches have indicated that the incidences of RA in different ethnic groups, geographical areas are different, and the RA prevalence is approximately 1% around the world (3, 4). It has been revealed that genetic susceptibilities, abnormal metabolic enzymes, aberrant immune response are involved in RA development (5, 6). Latterly, a number of single nucleotide polymorphisms (SNPs), the majority of which are located in the non-coding intervals, are gradually identified to be associated with the susceptibility of this disease according to genome-wide association studies (7, 8).

Long non-coding RNAs (lncRNAs), which are defined as RNAs longer than 200 nucleotides in length, have no or little protein-coding capacity (9). LncRNAs are reported to be involved in a variety of autoimmunity- and inflammation- related processes, and regulate gene expression in multiple mechanisms including alternative splicing, epigenetics, small RNA sponging (10, 11). Increasing studies have been performed to explore the potential role of lncRNAs on the pathogenesis of autoimmune diseases, such as RA and systemic lupus erythematosus (SLE) (12–14). Our recent study demonstrated that the lnc0640, lnc5150 expression levels were alternated among RA patients, and *lnc0640* rs13039216 TT genotype was statistically associated with RA susceptibility (14). Another previous study suggested that aberrant lncRNA expression level in peripheral blood mononuclear cells (PBMCs) could be a potential biomarker for RA diagnosing (13).

Recently, lncRNA ANRIL (antisense non-coding RNA in the INK4 locus) had attracted attention in autoimmune diseases, as it had been implicated in regulation of immune, inflammatory response (15). ANRIL expression was found to be regulated through STAT1 signaling pathway, which participated in immune regulation by induction of the pro-inflammatory cytokine TNF-γ (16). In addition, another study indicated that ANRIL expression level in PBMCs was decreased in RA by lncRNA array (13). Dendritic cell (DC) was a specific antigen presenting cell which link the innate and adaptive immune responses, and was thought to drive the activation of self-peptide-reactive inflammatory T cells, follicular helper T cells and consequently B cells for secreting autoantibodies in RA (17). Lnc-DC was a specialized, highly expressed lncRNA in DCs, and had the ability to regulate Th17 differentiation, DCs to stimulate T cell activation, and the production of interleukin 12 (IL-12) (9). LncRNA MALAT1 (metastasis-associated lung adenocarcinoma transcript-1) had been shown to play a role in the development of autoimmune diseases. SLE patients had increased MALAT1 level in PBMCs compared with normal individuals, and knockdown of MALAT-1 significantly suppressed IL-21 level in monocytes (18). In RA, Pan et al. found that knockdown of MALAT1 could inhibit the apoptosis of fibroblast-like synoviocytes (FLS) and lead to the activation of phosphoinositide 3-kinase (PI3K)/AKT signaling pathway (19). In another study, lncRNA ZFAS1 (zinc finger antisense 1) was shown to participate in RA-FLS migration and invasion by interacting with miR-27a and suppressing miR-27a expression, and ZFAS1 expression level was statistically evaluated in FLS of RA patients (20).

These studies demonstrated that ANRIL, lnc-DC, MALAT1, ZFAS1 might be involved in the occurrence and development of RA. However, no studies regarding the relationship between these lncRNAs genetic variation and RA have been reported. Thus, in the present study, we explored the associations of these lncRNAs genes SNPs with RA risk, as well as these lncRNAs expressions in PBMCs of RA patients and normal controls.

## Materials and Methods

### Patients and Normal Controls

In this study, case-control studies were performed in unrelated ethnic Han Chinese population. A total of 1,370 subjects including 660 RA patients and 710 normal controls were consecutively enrolled to investigate the association between *ANRIL, lnc-DC, MALAT1, ZFAS1* genes polymorphisms and RA susceptibility. Then, 120 RA patients and 120 normal controls were included to detect these lncRNAs expression levels. RA patients were selected from the First Affiliated Hospital of University of Science and Technology of China, and the First Affiliated Hospital of Anhui Medical University. The diagnosis of these patients was according to the 1987 American College of Rheumatology revised criteria (21). The normal controls, who were recruited from the healthy blood donors in the same region, did not have no a history of RA, or other inflammatory/autoimmune diseases, cancer. Disease Activity Score 28 (DAS 28) was used to evaluate RA disease activity (22). The demographic data of all subjects were collected, and the following clinical data of RA patients were retrieved from the medical records: complements 3 (C3), complements 4 (C4), erythrocyte sedimentation rate (ESR), C-reactive protein (CRP), anti-cyclic citrullinated peptide (anti-CCP), and rheumatoid factor (RF). After informed consent was obtained, peripheral blood samples and data were collected from RA patients and normal controls. This study protocol was approved by the Medical Ethics Committee of Anhui Medical University.

### SNP Selection, DNA Extraction, and Genotyping

The genetic and location information were obtained from two public databases, LNCipedia.org (v4.0) and Genome Browser Gateway (UCSC). We selected the tagSNPs with a minor allele frequency (MAF) ≥ 0.05 in CHB capturing all the common SNPs located in the chromosome locus transcribed into these lncRNAs (*ANRIL, lnc-DC, MALAT1, ZFAS1*) and their flanking 2,000 bp region through using genotype data of Han Chinese in Beijing from Ensembl genome browser 85 and CHBS_1000 g. The selection was conducted through linkage disequilibrium (LD) analysis with *r*^2^ threshold > 0.8 by using Haploview 4.0 software (Cambridge, MA, USA). In addition, the existing studies about these lncRNA genes polymorphisms were also reviewed. Finally, we selected six tagSNPs (rs1412830, rs7044859, rs944796, rs61271866, rs2518723, rs3217992) in *ANRIL, two* tagSNPs (rs7217280, rs10515177) in *lnc-DC*, five tagSNPs (rs619586, rs4102217, rs591291, rs11227209, rs35138901) in *MALAT1*, four tagSNPs (rs237742, rs73116127, rs6125607, rs6125608) in *ZFAS1* for genotyping in the present study.

The genomic DNA was extracted from the peripheral blood leukocytes by the Flexi Gene-DNA Kit (Qiagen, Valencia, CA). Improved multiple ligase detection reaction (iMLDR) genotyping assay, with technical support from the Center for Genetic & Genomic Analysis, Genesky Biotechnologies (Inc., Shanghai), was used for genotyping. Those individuals with 100% genotyping success rate for the above SNPs were included for final analysis.

### Quantitative Real-Time Reverse Transcription Polymerase Chain Reaction (qRT-PCR)

PBMCs were isolated from 5 ml anticoagulated peripheral blood, and stored at −80°C until processed. Total RNA in PBMCs was extracted with TRIzol Reagent (Invitrogen, Carlsbad, CA, USA) and the concentration of RNA was quantified using NanoDrop 2000 spectrophotometer (Thermo Scientific, USA). Then, the PrimeScriptTM RT reagent Kit (Takara Bio Inc., Japan) was used to reverse-transcribed total RNA into cDNA.

The expression levels of ANRIL, lnc-DC, MALAT1, ZFAS1 in PBMCs were detected by qRT-PCR with SYBR Green (SYBR Premix Ex Taq II, Takara Bio Inc., Japan). This experiment was performed on QuantStudio 12K Flex Real-Time PCR System (Applied Biosystems, Foster City, CA, USA), and according to the following cycle conditions: 95°C for 1 min, followed by 42 cycles at 95°C for 10 s, 60°C for 30 s and 72°C for 1 min. The relative expression levels of lncRNAs were calculated by comparison with housekeeping gene β-actin in the same sample as internal control, and expressed using 2^−ΔΔCt^ method normalized to endogenous control (23).

### Statistical Analysis

Statistical analysis was performed with the SPSS 23.0 (SPSS Inc., IL, USA). We performed Hardy-Weinberg equilibrium test by Chi-square (χ^2^) among normal controls. For the associations of genotype, allele distribution frequencies of each SNP with RA were estimated by logistic regression analyses. The lncRNAs levels were shown as median value and interquartile range, and the differences in lncRNAs levels between two groups, three groups were analyzed by Mann-Whitney *U*-test, Kruskal-Wallis *H*-test, respectively. The correlations between lncRNAs levels and several experimental indexes of RA patients were analyzed by Spearman rank correlation coefficient test. Dominant model, recessive mode was used for statistical analysis, and haplotype analysis was conducted with the SHEsis software (24). *P* value (two-sided) <0.05 was considered as statistically significant. False discovery rate (FRD) was used for multiple testing in SNP analysis.

## Results

### Association of lncRNAs Genes Polymorphisms With RA Susceptibility

We included 546 females and 114 males in RA patients for genotyping with a median age of 51, while there were 574 females and 136 males with a median age of 49 in normal controls. The observed genotype frequencies of rs7044859 was not conform to Hardy-Weinberg equilibrium, thus we excluded this SNP in finally analysis. The results of allele and genotype frequencies of all SNPs were summarized in Table 1.

**Table 1:** Genotypes and alleles frequencies of lncRNAs genes polymorphisms in RA patients and normal controls.

**SNP**	**Analyze model**		**RA (*N* = 660) *n* (%)**	**Control (*N* = 710) *n* (%)**	**Adjustment with sex and age**	
					*P* value^*^	***OR* (95% *CI*)**
***ANRIL***						
rs1412830	Genotypes	TT	13 (1.97)	3 (0.42)	0.017	0.214 (0.060–0.761)
		CT	119 (18.03)	139 (19.58)	0.564	1.084 (0.824–1.425)
		CC	528 (80.00)	568 (80.00)	Reference	
	Alleles	T	145 (10.98)	145 (10.21)	0.511	0.922 (0.722–1.176)
		C	1,175 (89.02)	1,275 (89.79)	Reference	
	Dominant model	CC	528 (80.00)	568 (80.00)	0.994	1.001 (0.766–1.307)
		TT+CT	132 (20.00)	142 (20.00)	Reference	
	Recessive model	TT	13 (1.97)	3 (0.42)	0.016	0.211 (0.059–0.750)
		CC+CT	647 (98.03)	707 (99.58)	Reference	
rs944796	Genotypes	GG	11 (1.67)	31 (4.37)	0.013	2.452 (1.211–4.962)
		GC	238 (36.06)	230 (32.39)	0.236	0.872 (0.695–1.094)
		CC	411 (62.27)	449 (63.24)	Reference	
	Alleles	G	260 (19.70)	292 (20.56)	0.572	1.055 (0.875–1.272)
		C	1,060 (80.30)	1,128 (79.44)	Reference	
	Dominant model	CC	411 (62.27)	449 (63.24)	0.598	1.061 (0.851–1.324)
		GG+GC	249 (37.73)	261 (36.76)	Reference	
	Recessive model	GG	11 (1.67)	31 (4.37)	0.008	2.574 (1.278–5.185)
		CC+GC	649 (98.33)	679 (95.63)	Reference	
rs61271866	Genotypes	AA	25 (3.79)	26 (3.66)	0.882	0.958 (0.542–1.692)
		TA	185 (28.03)	214 (30.14)	0.437	1.099 (0.867–1.392)
		TT	450 (68.18)	470 (66.20)	Reference	
	Alleles	A	235 (17.80)	266 (18.73)	0.529	0.940 (0.774–1.141)
		T	1,085 (82.20)	1,154 (81.27)	Reference	
	Dominant model	TT	450 (68.18)	470 (66.20)	0.498	0.924 (0.736–1.160)
		AA+TA	210 (31.82)	240 (33.80)	Reference	
	Recessive model	AA	25 (3.79)	26 (3.66)	0.811	0.933 (0.531–1.641)
		TT+TA	635 (96.21)	684 (96.34)	Reference	
rs2518723	Genotypes	TT	111 (16.82)	133 (18.73)	0.312	1.177 (0.858–1.613)
		CT	326 (49.39)	353 (49.72)	0.535	1.079 (0.848–1.372)
		CC	223 (33.79)	224 (31.55)	Reference	
	Alleles	T	548 (41.52)	619 (43.59)	0.256	1.092 (0.983–1.271)
		C	772 (58.48)	801 (56.41)	Reference	
	Dominant model	CC	223 (33.79)	224 (31.55)	0.393	0.906 (0.721–1.137)
		TT+CT	437 (66.21)	486 (68.45)	Reference	
	Recessive model	TT	111 (16.82)	133 (18.73)	0.413	0.890 (0.673–1.177)
		CC+CT	549 (83.18)	577 (81.27)	Reference	
rs3217992	Genotypes	TT	160 (24.24)	152 (21.41)	0.118	0.783 (0.576–1.064)
		CT	338 (51.21)	362 (50.99)	0.368	0.889 (0.687–1.149)
		CC	162 (24.55)	196 (27.61)	Reference	
	Alleles	T	658 (49.85)	666 (46.90)	0.123	0.889 (0.765–1.032)
		C	662 (50.15)	754 (53.10)	Reference	
	Dominant model	CC	162 (24.55)	196 (27.61)	0.206	1.170 (0.917–1.493)
		TT+CT	498 (75.45)	514 (72.39)	Reference	
	Recessive model	TT	160 (24.24)	152 (21.41)	0.199	0.846 (0.656–1.092)
		CC+CT	500 (75.76)	558 (78.59)	Reference	
***Lnc-DC***						
rs7217280	Genotypes	AA	3 (0.45)	4 (0.56)	0.849	1.160 (0.253–5.314)
		GA	52 (7.88)	77 (10.85)	0.084	1.388 (0.957–2.014)
		GG	605 (91.67)	629 (88.59)	Reference	
	Alleles	A	58 (4.39)	85 (5.99)	0.062	1.385 (0.984–1.951)
		G	1,262 (95.61)	1,335 (94.01)	Reference	
	Dominant model	GG	605 (91.67)	629 (88.59)	0.085	0.727 (0.506–1.045)
		AA+GA	55 (8.33)	81 (11.41)	Reference	
	Recessive model	AA	3 (0.45)	4 (0.56)	0.881	1.123 (0.245–5.146)
		GG+GA	657 (99.55)	706 (99.44)	Reference	
rs10515177	Genotypes	GG	4 (0.61)	5 (0.70)	0.870	1.118 (0.294–4.249)
		GA	94 (14.24)	117 (16.48)	0.330	1.159 (0.861–1.560)
		AA	562 (85.15)	588 (82.82)	Reference	
	Alleles	G	102 (7.73)	127 (8.94)	0.251	1.173 (0.893–1.540)
		A	1,218 (92.27)	1,293 (91.06)	Reference	
	Dominant model	AA	562 (85.15)	588 (82.82)	0.327	0.864 (0.645–1.157)
		GG+GA	98 (14.85)	122 (17.18)	Reference	
	Recessive model	GG	4 (0.61)	5 (0.70)	0.896	1.093 (0.288–4.151)
		AA+GA	656 (99.39)	705 (99.30)	Reference	
***MALAT1***						
rs619586	Genotypes	GG	6 (0.91)	4 (0.56)	0.350	0.544 (0.151–1.951)
		GA	111 (16.82)	113 (15.92)	0.628	0.931 (0.698–1.243)
		AA	543 (82.27)	593 (83.52)	Reference	
	Alleles	G	123 (9.32)	121 (8.52)	0.464	0.906 (0.697–1.179)
		A	1,197 (90.68)	1,299 (91.48)	Reference	
	Dominant model	AA	543 (82.27)	593 (83.25)	0.517	1.098 (0.827–1.458)
		GG+GA	117 (17.73)	117 (16.48)	Reference	
	Recessive model	GG	6 (0.91)	4 (0.56)	0.359	0.550 (0.153–1.973)
		AA+GA	654 (99.09)	706 (99.44)	Reference	
rs4102217	Genotypes	CC	20 (3.03)	13 (1.83)	0.306	0.688 (0.337–1.408)
		CG	154 (23.33)	205 (28.87)	0.020	1.340 (1.047–1.713)
		GG	486 (73.64)	492 (69.30)	Reference	
	Alleles	C	194 (14.70)	231 (16.27)	0.257	1.128 (0.916–1.388)
		G	1,126 (85.30)	1,189 (83.73)	Reference	
	Dominant model	GG	486 (73.64)	492 (69.30)	0.053	0.791 (0.624–1.003)
		CC+CG	174 (26.36)	218 (30.70)	Reference	
	Recessive model	CC	20 (3.03)	13 (1.83)	0.216	0.638 (0.313–1.300)
		GG+CG	640 (96.97)	697 (98.17)	Reference	
rs591291	Genotypes	TT	124 (18.79)	132 (18.59)	0.496	1.113 (0.818–1.513)
		CT	298 (45.15)	347 (48.87)	0.125	1.207 (0.949–1.534)
		CC	238 (36.06)	231 (32.53)	Reference	
	Alleles	T	546 (41.36)	611 (43.03)	0.378	1.071 (0.920–1.246)
		C	774 (58.64)	809 (56.97)	Reference	
	Dominant model	CC	238 (36.06)	231 (32.53)	0.153	0.848 (0.677–1.063)
		CT+TT	422 (63.94)	479 (67.46)	Reference	
	Recessive model	TT	124 (18.79)	132 (18.59)	0.979	0.996 (0.757–1.310)
		CT+CC	536 (81.21)	578 (81.41)	Reference	
rs11227209	Genotypes	GG	3 (0.45)	3 (0.42)	0.880	0.883 (0.176–4.420)
		CG	71 (10.76)	79 (11.13)	0.773	1.052 (0.747–1.480)
		CC	586 (88.79)	628 (88.45)	Reference	
	Alleles	G	77 (5.83)	85 (5.99)	0.866	1.028 (0.748–1.412)
		C	1,243 (94.17)	1,335 (94.01)	Reference	
	Dominant model	CC	586 (88.79)	628 (88.45)	0.799	0.957 (0.684–1.339)
		CG+GG	74 (11.21)	82 (11.55)	Reference	
	Recessive model	GG	3 (0.45)	3 (0.42)	0.874	0.878 (0.176–4.393)
		CG+CC	657 (99.55)	707 (99.58)	Reference	
rs35138901	Genotypes	CC	4 (0.61)	2 (0.28)	0.469	0.532 (0.097–2.933)
		CT	93 (14.09)	115 (16.20)	0.252	1.191 (0.883–1.606)
		TT	563 (85.30)	593 (83.52)	Reference	
	Alleles	C	101 (7.65)	119 (8.38)	0.483	1.104 (0.837–1.445)
		T	1,219 (92.35)	1,301 (91.62)	Reference	
	Dominant model	TT	563 (85.30)	593 (83.52)	0.312	0.859 (0.639–1.154)
		CT+CC	97 (14.70)	117 (16.48)	Reference	
	Recessive model	CC	4 (0.61)	2 (0.28)	0.450	0.518 (0.094–2.852)
		CT+TT	656 (99.39)	708 (99.72)	Reference	
***ZFAS1***						
rs237742	Genotypes	TT	91 (13.79)	104 (14.65)	0.994	0.999 (0.717–1.391)
		CT	322 (48.79)	320 (45.07)	0.212	0.863 (0.685–1.088)
		CC	247 (37.42)	286 (40.28)	Reference	
	Alleles	T	504 (38.18)	528 (37.18)	0.590	0.958 (0.821–1.119)
		C	816 (61.82)	892 (62.82)	Reference	
	Dominant model	CC	247 (37.42)	286 (40.28)	0.309	1.121 (0.900–1.395)
		CT+TT	413 (62.58)	424 (59.72)	Reference	
	Recessive model	TT	91 (13.79)	104 (14.65)	0.611	1.083 (0.797–1.470)
		CT+CC	569 (86.21)	606 (85.35)	Reference	
rs73116127	Genotypes	AA	1 (0.15)	3 (0.42)	0.384	2.739 (0.283–26.506)
		GA	109 (16.52)	133 (18.73)	0.294	1.162 (0.878–1.538)
		GG	550 (83.33)	574 (80.85)	Reference	
	Alleles	A	111 (8.41)	139 (9.79)	0.211	1.182 (0.910–1.535)
		G	1,209 (91.59)	1,281 (90.21)	Reference	
	Dominant model	GG	550 (83.33)	574 (80.85)	0.253	0.850 (0.643–1.123)
		AA+GA	110 (16.67)	136 (19.15)	Reference	
	Recessive model	AA	1 (0.15)	3 (0.42)	0.398	2.661 (0.275–25.738)
		GG+GA	659 (99.85)	707 (99.58)	Reference	
rs6125607	Genotypes	TT	74 (11.21)	48 (6.76)	0.007	0.576 (0.387–0.857)
		CT	277 (41.97)	310 (43.66)	0.978	1.003 (0.801–1.256)
		CC	309 (46.82)	352 (49.58)	Reference	
	Alleles	T	425 (32.20)	406 (28.59)	0.040	0.843 (0.716–0.992)
		C	895 (67.80)	1,014 (71.41)	Reference	
	Dominant model	CC	309 (46.82)	352 (49.58)	0.407	1.095 (0.884–1.356)
		TT+CT	351 (53.18)	358 (50.42)	Reference	
	Recessive model	TT	74 (11.21)	48 (6.76)	0.005	0.576 (0.393–0.844)
		CC+TC	586 (88.78)	662 (93.23)	Reference	
rs6125608	Genotypes	GG	9 (1.36)	11 (1.55)	0.716	1.181 (0.483–2.890)
		GA	125 (18.94)	158 (22.25)	0.153	1.213 (0.931–1.582)
		AA	526 (79.70)	541 (76.20)	Reference	
	Alleles	G	143 (10.83)	180 (12.68)	0.135	1.195 (0.946–1.509)
		A	1,177 (89.17)	1,240 (87.32)	Reference	
	Dominant model	AA	526 (79.70)	541 (76.20)	0.147	0.826 (0.638–1.069)
		GG+GA	134 (20.30)	169 (23.80)	Reference	
	Recessive model	GG	9 (1.36)	11 (1.55)	0.780	1.136 (0.465–2.775)
		AA+GA	651 (98.64)	699 (98.45)	Reference	

* *After FDR correction, no P value was statistically significant (all P > 0.05)*.

In *ANRIL* gene, the rs1412830 TT genotype frequency was significantly increased in RA patients in comparison to normal controls, while the rs944796 GG genotype frequency was significantly decreased (TT vs. CC: *P* = 0.017; GG vs. CC: *P* = 0.013, respectively). In addition, an increased risk of rs1412830 variant, as well as a decreased risk of rs944796, was observed under the recessive model (TT vs. CC+CT: *P* = 0.016; GG vs. CC+GC: *P* = 0.008, respectively). However, these significant associations were disappeared after multiple testing by FDR correction (all *P* > 0.05). Comparing the genotype and allele frequencies of the ZFAS1 rs6125607 polymorphism among RA patients and normal controls, we found that TT genotype and T allele frequencies were significantly higher in RA patients than normal controls (TT vs. CC: *P* = 0.007; T vs. C: *P* = 0.040, respectively), and an increased risk of rs6125607 polymorphism existed in recessive model (TT vs. CC+TC: *P* = 0.005). After FDR correction, these differences were not statistically significant (TT vs. CC: *P* = 0.181; T vs. C: *P* = 0.496, TT vs. CC+TC: *P* = 0.080, respectively). Similarly, no significant associations between *lnc-DC, MALAT1* genes polymorphism and RA susceptibility were found (Table 1).

To examine the potential genetic association between the genotype, allele frequencies of *ANRIL, lnc-DC, MALAT1, ZFAS1* genes and anti-CCP, RF in RA patients, we performed a case-only analysis (Table S1). In *ANRIL* gene, the rs944796 G allele, rs2518723 T allele frequencies were significantly increased in RA patients with anti-CCP-positive when compared to patients with anti-CCP-negative (all *P* < 0.05), while rs3217992 T allele frequency was reduced (*P* = 0.039) (Table 2). No significant differences existed in allele and genotype frequencies of *lnc-DC, MALAT1, ZFAS1* genes.

**Table 2:** The positive findings of associations between *ANRIL* gene polymorphisms and anti-CCP of RA patients.

**SNP**	**Allele**	**Clinical features**	**Group**	**Alleles** ***n*** **(%)**		***P* value**
	***(M/m)***			***M***	***m***	
rs944796	C/G	Anti-CCP	Positive	853 (79.28)	223 (20.72)	**0.039**
			Negative	143 (86.14)	23 (13.86)	
rs2518723	C/T	Anti-CCP	Positive	615 (57.16)	461 (42.84)	**0.039**
			Negative	109 (65.66)	57 (34.34)	
rs3217992	C/T	Anti-CCP	Positive	553 (51.39)	523 (48.61)	**0.039**
			Negative	71 (42.77)	95 (57.23)	

*Bold value means P < 0.05*.

### Haplotype Analysis

Six main haplotypes (CCATC, CCTCC, CCTCT, CCTTC, CGTTC, TCATC) for *ANRIL*, three main haplotypes (AG, GA, GG) for lnc-DC, six main haplotypes (ACTCT, AGCCT, AGTCC, AGTCT, GGTCT, GGTGT) for *MALAT1* and five main haplotypes (CACA, CGCA, CGCG, CGTA, TGCA) for *ZFAS1* were detected by SHEsis software (Table 3). The results demonstrated that the haplotype CGTA frequency was significantly higher in RA patients than normal controls (*OR* = 1.191, 95% CI: 1.012–1.402, *P* = 0.036).

**Table 3:** Haplotype analysis of lncRNA genes in RA patients and controls.

**Haplotype**	**RA patients [*n*(%)]**	**Controls [*n*(%)]**	***P* value**	***OR* (95% CI)**
***ANRIL*** **rs1412830- rs944796- rs61271866- rs2518723- rs3217992**				
CCATC	91.88 (7.0)	125.21 (8.8)	0.077	0.776 (0.586–1.028)
CCTCC	105.84 (8.0)	108.60 (7.6)	0.692	1.058 (0.800–1.400)
CCTCT	614.30 (46.5)	621.58 (43.8)	0.109	1.135 (0.972–1.324)
CCTTC	116.57 (8.8)	128.95 (9.1)	0.847	0.974 (0.749–1.268)
CGTTC	190.02 (14.4)	220.98 (15.6)	0.419	0.916 (0.742–1.132)
TCATC	122.50 (9.3)	135.90 (9.6)	0.824	0.971 (0.751–1.256)
***Lnc-DC*** **rs7217280- rs10515177**				
AG	58.00 (4.4)	85.00 (6.0)	0.061	0.722 (0.512–1.017)
GA	1,218.00 (92.3)	1,293.00 (91.1)	0.250	1.173 (0.893–1.540)
GG	44.00 (3.3)	42.00 (3.0)	0.573	1.131 (0.736–1.738)
***MALAT1*** **rs619586- rs4102217- rs591291- rs11227209- rs35138901**				
ACTCT	192.89 (14.6)	227.78 (16.0)	0.297	0.895 (0.727–1.103)
AGCCT	769.36 (58.3)	804.34 (56.6)	0.393	1.069 (0.918–1.244)
AGTCC	96.61 (7.3)	115.61 (8.1)	0.419	0.890 (0.672–1.180)
AGTCT	133.58 (10.1)	147.87 (10.4)	0.796	0.968 (0.756–1.239)
GGTCT	45.92 (3.5)	32.65 (2.3)	0.065	1.531 (0.971–2.413)
GGTGT	75.01 (5.7)	83.88 (5.9)	0.799	0.959 (0.696–1.322)
***ZFAS1*** **rs237742- rs73116127- rs6125607- rs6125608**				
CACA	111.00 (8.4)	136.66 (9.6)	0.259	0.860 (0.661–1.118)
CGCA	137.00 (10.4)	170.49 (12.0)	0.170	0.846 (0.667–1.074)
CGCG	143.00 (10.8)	177.70 (12.5)	0.164	0.847 (0.670–1.070)
CGTA	425.00 (32.2)	403.76 (28.4)	**0.036**	1.191 (1.012–1.402)
TGCA	504.00 (38.2)	527.90 (37.2)	0.622	1.040 (0.891–1.214)

*Frequency < 0.03 in both controls and RA patients has been dropped. Bold value means P < 0.05*.

### LncRNAs Expression Levels in PBMCs From RA Patients and Normal Controls

We further analyzed the association of ANRIL, lnc-DC, MALAT1, ZFAS1 levels with RA patients by qRT-PCR. As shown in Table 4, the expression levels of ANRIL, lnc-DC, MALAT1, ZFAS1 in PBMCs were significantly reduced in RA patients than normal controls (all *P* < 0.001). However, the differences in these lncRNAs levels between anti-CCP-positive RA patients and anti-CCP-negative RA patients, as well as RA patients with RF-positive and RF-negative RA patients, were not statistically significant.

**Table 4:** Comparison of lncRNAs expression level in PBMCs between different subgroups.

**Group**	**Number**	**ANRIL**	***P* value**	**Lnc-DC**	***P* value**	**MALAT1**	***P* value**	**ZFAS1**	***P* value**
RA patients	120	0.605 (0.382, 0.849)	<0.001	0.378 (0.269, 0.586)	<0.001	0.409 (0.257, 0.533)	<0.001	0.458 (0.352, 0.646)	<0.001
Normal controls	120	0.853 (0.612, 1.147)		0.818 (0.537, 1.166)		0.932 (0.627, 1.228)		0.870 (0.625, 1.161)	
RA patients with anti-CCP-positive	99	0.603 (0.404, 0.868)	0.866	0.387 (0.264, 0.591)	0.920	0.418 (0.250, 0.543)	0.681	0.469 (0.362, 0.635)	0.926
RA patients with anti-CCP-negative	21	0.619 (0.350, 0.823)		0.358 (0.284, 0.577)		0.372 (0.270, 0.489)		0.391 (0.337, 0.676)	
RA patients with anti-RF-positive	101	0.629 (0.404, 0.870)	0.210	0.344 (0.268, 0.561)	0.453	0.415 (0.251, 0.575)	0.563	0.469 (0.352, 0.642)	0.997
RA patients with anti-RF-negative	19	0.510 (0.360, 0.675)		0.436 (0.273, 0.614)		0.379 (0.306, 0.474)		0.444 (0.349, 0.682)	

The correlation of ANRIL, lnc-DC, MALAT1, ZFAS1 expression levels with clinical parameters, disease activity of RA patients were also analyzed, and the results shown that the expression level of ZFAS1 was negatively associated with CRP in RA patients (*P* = 0.002). However, there were no significant correlations of these lncRNAs levels with DAS28 of RA patients (Table 5). The potential influence of main medical therapies including glucocorticoids, disease-modifying antirheumatic drugs (DMARDs), biologics on lncRNAs expression levels in RA patients were assessed in this study. Similarly, no significant association was found (Table 6).

**Table 5:** Association of lncRNAs expression levels with clinical parameters, disease activity of RA patients.

**Parameters**	**Number**	**ANRIL**		**Lnc-DC**		**MALAT1**		**ZFAS1**	
		***r_***s***_***	***P* value**	***r_s_***	***P* value**	***r_**s**_***	***P* value**	***r_***s***_***	***P* value**
C3	107	−0.035	0.719	−0.054	0.583	0.077	0.431	−0.100	0.305
C4	106	−0.122	0.213	−0.027	0.781	0.094	0.339	−0.020	0.840
ESR	118	0.035	0.705	0.034	0.712	0.069	0.457	−0.090	0.334
CRP	118	−0.038	0.682	−0.094	0.313	−0.178	0.054	−0.278	0.002
DAS28	118	0.071	0.444	0.139	0.132	−0.078	0.399	−0.036	0.695

**Table 6:** Association of these lncRNAs expression levels with medical therapy of RA patients.

**Group**	**Number**	**ANRIL level**	***P* value**	**Lnc-DC level**	***P* value**	**MALAT1 level**	***P* value**	**ZFASA level**	***P* value**
Glucocorticoids			0.302		0.764		0.340		0.500
NA	30	0.516 (0.344, 0.948)		0.445 (0.256, 0.608)		0.450 (0.259, 0.581)		0.452 (0.355, 0.597)	
≤7.5 mg/d	28	0.573 (0.323, 0.831)		0.325 (0.250, 0.512)		0.437 (0.240, 0.561)		0.464 (0.299, 0.614)	
>7.5 mg/d	62	0.634 (0.451, 0.834)		0.348 (0.278, 0.580)		0.389 (0.374, 0.703)		0.462 (0.374, 0.703)	
DMARDs			0.532		0.232		0.366		0.257
No	39	0.619 (0.428, 0.901)		0.414 (0.269, 0.614)		0.404 (0.226, 0.539)		0.410 (0.349, 0.575)	
Yes	81	0.587 (0.350, 0.835)		0.338 (0.265, 0.540)		0.418 (0.273, 0.533)		0.471 (0.361, 0.679)	
Biologics			0.489		0.423		0.211		0.095
No	111	0.603 (0.399, 0.838)		0.377 (0.266, 0.570)		0.408 (0.256, 0.533)		0.455 (0.345, 0.618)	
Yes	9	0.760 (0.291, 1.120)		0.461 (0.289, 0.687)		0.432 (0.395, 0.588)		0.632 (0.392, 0.902)	

### Associations Between lncRNAs Genes Polymorphisms With Their Levels in RA Patients

To examine the associations between the respective genotype frequencies of these lncRNAs genes with their expression levels in RA patients, we included 65 patients for analysis. However, there were no significant differences regarding these lncRNAs expression levels between their disparate genotypes of RA patients (Table 7).

**Table 7:** Association between lncRNA levels with their respective genotype in RA patients.

***ANRIL* SNPs**	**Genotype**	**Number**	***ANRIL* level**	***P* value**
rs1412830	CC	49	0.542 (0.404, 0.742)	0.210
	CT	14	0.418 (0.267, 0.672)	
	TT	2	0.913 (0.504, 1.322)	
rs944796	CC	36	0.585 (0.371, 0.798)	0.437
	GC	27	0.493 (0.360, 0.786)	
	GG	2	0.325 (0.088, 0.562)	
rs61271866	TT	44	0.562 (0.404, 0.764)	0.251
	TA	19	0.497 (0.274, 0.641)	
	AA	2	0.913 (0.504, 1.323)	
rs2518723	CC	21	0.603 (0.414, 0.975)	0.329
	CT	28	0.520 (0.339, 0.646)	
	TT	16	0.476 (0.335, 0.818)	
rs3217992	CC	21	0.459 (0.296, 0.647)	0.219
	CT	29	0.582 (0.472, 0.839)	
	TT	15	0.587 (0.404, 0.955)	
***Lnc-DC*** **SNPs**	**Genotype**	**Number**	**Lnc-DC level**	***P*** **value**
rs7217280	GG	63	0.378 (0.282, 0.603)	0.649
	GA	2	0.458 (0.431, 0.484)	
	AA	0		
rs10515177	GG	58	0.383 (0.289, 0.600)	0.703
	GA	7	0.431 (0.269, 0.629)	
	AA	0		
***MALAT1*** **SNPs**	**Genotype**	**Number**	**MALAT1 level**	***P*** **value**
rs619586	AA	55	0.415 (0.253, 0.530)	0.167
	GA	10	0.285 (0.128, 0.473)	
	GG	0		
rs4102217	GG	48	0.337 (0.214, 0.486)	0.064
	CG	13	0.477 (0.381, 0.634)	
	CC	4	0.463 (0.380, 0.851)	
rs591291	CC	24	0.416 (0.257, 0.502)	0.905
	CT	29	0.415 (0.199, 0.508)	
	TT	12	0.389 (0.155, 0.617)	
rs11227209	CC	59	0.415 (0.245, 0.530)	0.267
	CG	6	0.285 (0.181, 0.473)	
	GG	0		
rs35138901	TT	56	0.418 (0.257, 0.526)	0.068
	CT	9	0.193 (0.161, 0.454)	
	CC	0		
***ZFAS1*** **SNPs**	**Genotype**	**Number**	**ZFAS1 level**	***P*** **value**
rs237742	CC	31	0.471 (0.376, 0.584)	0.392
	CT	30	0.394 (0.300, 0.605)	
	TT	4	0.423 (0.359, 0.783)	
rs73116127	GG	51	0.444 (0.362, 0.6001)	0.342
	GA	14	0.377 (0.325, 0.529)	
	AA	0		
rs6125607	CC	25	0.398 (0.351, 0.516)	0.375
	CT	31	0.434 (0.316, 0.618)	
	TT	9	0.523 (0.419, 0.655)	
rs6125608	AA	50	0.438 (0.353, 0.600)	0.998
	GA	14	0.428 (0.344, 0.652)	
	CC	1	0.444	

*Median (interquartile range)*.

## Discussion

To date, the exact pathogenic mechanism of RA remains largely unknown, although several pro-inflammatory cytokines including tumor necrosis factor-α (TNF-α), IL-6, IL-1b have been reported to related to the occurrence of RA (25–27). Previous studies have shown that lncRNAs had distinct and specific roles in the activation and differentiation modulating of immune cell, and lncRNAs played an important role in autoimmune diseases (28). A study detected lncRNA transcription in CD14+ monocytes isolated from peripheral blood cells of RA patients before and after anti-IL-6R (tocilizumab) or anti-TNF-α (adalimumab) therapy by a microarray-based experiment. They observed that 7,419 lncRNAs expression levels were altered by either IL-6 or TNF-α, 85 of which exhibited were significant changed (29). These results suggested that lncRNAs were very important in the molecular pathophysiology of RA. In the present study, our results demonstrated that lower expression levels of ANRIL, lnc-DC, MALAT1, ZFAS1 existed in RA patients, and *ANRIL, lnc-DC, MALAT1, ZFAS1* genes were not related to RA susceptibility.

*ANRIL* gene was located in the chromosome 9p21 region, and it was the well-defined genetic risk locus related to several diseases such as coronary artery disease (CAD), diabetes, and breast cancer (30–32). Our results implied that *ANRIL* rs1412830, rs944796 variant might associated with RA susceptibility, while the significant associations were disappeared after multiple testing. However, we found that *ANRIL* rs944796 G, rs2518723 T, rs3217992 T allele frequencies were significantly associated with anti-CCP in RA patients, this suggested to us that *ANRIL* gene variation might be involved in the RA development. In addition, disease-associated SNPs resided in this region had been reported to change the expression of ANRIL, demonstrating that altered ANRIL expression might be involved in predisposition to these disorders (33). Two SNPs (rs10757278 and rs1333045) in *ANRIL*, which had been highlighted as potential causal variants for the association with CAD, were reported to be associated with abnormal ANRIL expression level in Peripheral blood (34, 35). Moreover, our results demonstrated that compared with normal controls, ANRIL expression level was significantly decreased in PBMC from RA patients. We further explored the influence of the five SNPs on ANRIL level in PBMC from RA patients, unfortunately, there were no significant differences regarding ANRIL level between disparate genotypes of these SNPs.

In a previous study, the authors discovered a new lncRNA (named lnc-DC) located on chromosome 17 region, which near signal transducer and activator of transcription 3 (*STAT3*) gene (36). There were increasing researches to explore the contribution of lnc-DC in autoimmune diseases. Shaker et al. found that serum level of lnc-DC in multiple sclerosis (MS) patients were significantly increased, and serum lnc-DC level maybe used to as a potential novel biomarkers for MS diagnosis (37). One of our recent studies shown that the lnc-DC expression level was significantly decreased in PBMCs from SLE patients than controls, while *lnc-DC* rs10515177 variant was not associated with SLE susceptibility (38). Similarly, the lnc-DC expression level was significantly lower in PBMC from RA patients than normal controls in the present study. We also explore the potential association of two SNP (rs7217280, rs10515177) in *lnc-DC* with RA susceptibility, however, no significant relationship was found. Our study provided the first evidence that lnc-DC might be involved in the development of RA, and the specific roles of *lnc-DC* genetic variation in pathophysiology of RA need to be further explored.

MALAT1 was expressed on chromosome 11q13, and widely expressed in multiple normal tissues such as reproductive, endocrine and immune systems with an important role in autoimmune diseases including RA, SLE, MS (18, 19, 37, 39). Quercetin is a dietary antioxidant, which has been shown to be effective in the treatment of arthritis in pre-clinical studies, and Pan et al. tried to analyze the mechanisms responsible for the quercetin-induced FLS apoptosis in RA patients (19). Their data indicated that quercetin induced FLS apoptosis in RA patients via upregulating MALAT1, and MALAT1 promoted apoptosis by inhibiting the activation of the phosphoinositide 3-kinase (PI3K)/AKT pathway. In this study, decreased MALAT1 expression level in PBMC from RA patients was firstly reported, while *MALAT1* genetic variation was not correlated with RA risk. Our findings provided new evidence that MALAT1 might be involved in RA development.

ZFAS1, located at chromosomal band 20q13.13, was reported as an important player to regulate the development of human cancers including glioma, lung, ovary, gastric, and breast cancer (40–42). In addition, ZFAS1 was found to promote chondrocytes proliferation, migration, and inhibit apoptosis and matrix synthesis in osteoarthritis (OA), and ZFAS1 expression level was downregulated in OA chondrocytes in comparison to mild chondrocytes (43). Another study by Xiao et al. also found more than five times ZFAS1 level in the healthy appearing area of cartilage compared with the pathology area in human knee osteoarthritis (44). Similarly, our results demonstrated that the expression of ZFAS1 in PBMCs was significantly reduced in RA patients than normal controls, and associated with CRP in RA. In the present study, we also analyzed the potential relationship between rs237742, rs73116127, rs6125607, rs6125608 variants in *ZFAS1* and genetic susceptibility to RA, and no difference achieved statistical significance. However, haplotype analysis implied that the haplotype CGTA frequency for *ZFAS1* was significantly higher in RA patients than normal controls. These founding would help improve our understanding of the roles of *ZFAS1* genetic variants in the pathogenesis of RA.

In conclusion, our study provided the first evidence that *ANRIL, lnc-DC, MALAT1, ZFAS1* genes polymorphisms might not be associated with RA susceptibility in the Chinese population. However, several SNPs in *ANRIL* were related to anti-CCP in RA. In addition, alternations of ANRIL, lnc-DC, MALAT1, ZFAS1 levels and significant correlations of ZFAS1 level with CRP in RA patient demonstrated that these lncRNAs might be regarded as an auxiliary biomarker for RA diagnosis, as well as used to distinguish RA serotypes.

However, some limitations existed in our study should be acknowledged. Firstly, this study does not eliminate the potential influence of ethnic background, environmental factor. Secondly, we are not able to assess the associations between these lncRNAs levels and disease severity, clinical variables, and therapeutic schedule of RA patients over a long period in this case-control study. Finally, our sample size may not be sufficient, and lead to the low power of this study. Hence, replication studies with larger sample size, different ethnic populations are awaited to further explore the exact role of these lncRNAs in RA.

## Ethics Statement

This study was approved by the Ethical Committee of Anhui Medical University (Hefei, Anhui, China). All the study subjects provided informed consent to participate in this study. All studies on humans described in the present manuscript were carried out with the approval of the responsible ethics committee and in accordance with national law and the Helsinki Declaration of 1975 (in its current, revised form).

## Author Contributions

T-PZ, H-FP, and D-QY designed the study. T-PZ and B-QZ conducted the experiment. S-ST and Y-GF performed the statistical analyses. T-PZ drafted the manuscript. X-ML participated in the collection of samples. H-FP and D-QY contributed to manuscript revision. All the authors approved the final submitted version.

### Conflict of Interest

The authors declare that the research was conducted in the absence of any commercial or financial relationships that could be construed as a potential conflict of interest.
