# Supplementary material for: Long Non-coding RNAs Genes Polymorphisms and Their Expression Levels in Patients With Rheumatoid Arthritis
Source: Front Immunol. 2019 Oct 31;10:2529. doi: 10.3389/fimmu.2019.02529 (PMC6834534; doi:10.3389/fimmu.2019.02529)
Supplement: Supplementary file 1 [file Table_1.DOC]

Table S1 Associations between lncRNA genes polymorphisms and anti-CCP, RF of RA patients

| SNP | Allele | Clinical features | Group | Genotypes n (%) | | | *P* value | Alleles n (%) | | *P* value |
| --- | --- | --- | --- | --- | --- | --- | --- | --- | --- | --- |
|  | (M/m) |  |  | MM | Mm | mm |  | M | m |  |
| *ANRIL* | | | | | | |  |  | | |
| rs1412830 | C/T | anti-CCP | Positive | 430(79.93) | 97(18.03) | 11(2.04) | 0.857 | 957(88.94) | 119(11.06) | 0.753 |
|  |  |  | Negative | 68(81.93) | 13(15.66) | 2(2.41) |  | 149(89.76) | 17(10.24) |  |
|  |  | RF | Positive | 426(80.38) | 92(17.36) | 12(2.26) | 0.616 | 944(89.06) | 116(10.94) | 0.799 |
|  |  |  | Negative | 89(80.18) | 21(18.92) | 1(0.90) |  | 199(89.64) | 23(10.36) |  |
| rs944796 | C/G | anti-CCP | Positive | 326(60.59) | 201(37.36) | 11(2.04) | 0.077 | 853(79.28) | 223(20.72) | **0.039** |
|  |  |  | Negative | 60(72.29) | 23(27.71) | 0 |  | 143(86.14) | 23(13.86) |  |
|  |  | RF | Positive | 327(61.70) | 193(36.42) | 10(1.89) | 0.441 | 847(79.91) | 213(20.09) | 0.241 |
|  |  |  | Negative | 75(67.57) | 35(31.53) | 1(0.90) |  | 185(83.33) | 37(16.67) |  |
| rs61271866 | T/A | anti-CCP | Positive | 362(67.29) | 156(29.00) | 20(3.71) | 0.610 | 880(81.78) | 196(18.22) | 0.674 |
|  |  |  | Negative | 59(71.08) | 20(24.10) | 4(4.82) |  | 138(83.13) | 28(16.87) |  |
|  |  | RF | Positive | 363(68.49) | 146(27.55) | 21(3.96) | 0.953 | 872(82.26) | 188(17.74) | 0.920 |
|  |  |  | Negative | 75(67.57) | 32(28.83) | 4(3.60) |  | 182(81.98) | 40(18.02) |  |
| rs2518723 | C/T | anti-CCP | Positive | 171(31.78) | 273(50.74) | 94(17.47) | 0.108 | 615(57.16) | 461(42.84) | **0.039** |
|  |  |  | Negative | 35(42.17) | 39(46.99) | 9(10.84) |  | 109(65.66) | 57(34.34) |  |
|  |  | RF | Positive | 178(33.58) | 266(50.19) | 86(16.23) | 0.795 | 622(58.68) | 438(41.32) | 0.557 |
|  |  |  | Negative | 41(36.94) | 53(47.75) | 17(15.32) |  | 135(60.81) | 87(39.19) |  |
| rs3217992 | C/T | anti-CCP | Positive | 135(25.09) | 283(52.60) | 120(22.30) | 0.068 | 553(51.39) | 523(48.61) | **0.039** |
|  |  |  | Negative | 16(19.28) | 39(46.99) | 28(33.73) |  | 71(42.77) | 95(57.23) |  |
|  |  | RF | Positive | 130(24.53) | 275(51.89) | 125(23.58) | 0.444 | 535(50.47) | 525(49.53) | 0.220 |
|  |  |  | Negative | 23(20.72) | 56(50.45) | 32(28.83) |  | 102(45.95) | 120(54.05) |  |
| *Lnc-DC* |  |  |  |  |  |  |  |  |  |  |
| rs7217280 | G/A | anti-CCP | Positive | 494(91.82) | 42(7.80) | 2(0.37) | 0.581 | 1030(95.72) | 46(4.28) | 0.504 |
|  |  |  | Negative | 75(90.36) | 7(8.43) | 1(1.20) |  | 157(94.58) | 9(5.42) |  |
|  |  | RF | Positive | 488(92.08) | 41(7.74) | 1(0.19) | 0.068 | 1017(95.94) | 43(4.06) | 0.139 |
|  |  |  | Negative | 99(89.19) | 10(9.01) | 2(1.80) |  | 208(93.69) | 14(6.31) |  |
| rs10515177 | A/G | anti-CCP | Positive | 460(85.50) | 75(13.94) | 3(0.56) | 0.605 | 995(92.47) | 81(7.53) | 0.346 |
|  |  |  | Negative | 68(81.93) | 14(16.87) | 1(1.20) |  | 150(90.36) | 16(9.64) |  |
|  |  | RF | Positive | 453(85.47) | 75(14.15) | 2(0.38) | 0.208 | 981(92.55) | 79(7.45) | 0.311 |
|  |  |  | Negative | 92(82.88) | 17(15.32) | 2(1.80) |  | 201(90.54) | 21(9.46) |  |
| *MALAT1* |  |  |  |  |  |  |  |  |  |  |
| rs619586 | A/G | anti-CCP | Positive | 440(81.78) | 92(17.10) | 6(1.12) | 0.624 | 972(90.33) | 104(9.67) | 0.614 |
|  |  |  | Negative | 69(83.13) | 14(16.87) | 0 |  | 152(91.57) | 14(8.43) |  |
|  |  | RF | Positive | 434(81.89) | 90(16.98) | 6(1.13) | 0.473 | 958(90.38) | 102(9.62) | 0.359 |
|  |  |  | Negative | 94(84.68) | 17(15.32) | 0 |  | 205(92.34) | 17(7.66) |  |
| rs4102217 | G/C | anti-CCP | Positive | 402(74.72) | 117(21.75) | 19(3.53) | 0.068 | 921(85.60) | 155(14.40) | 0.823 |
|  |  |  | Negative | 58(69.88) | 25(30.12) | 0 |  | 141(84.94) | 25(15.06) |  |
|  |  | RF | Positive | 399(75.28) | 116(21.89) | 15(2.83) | 0.242 | 914(86.23) | 146(13.77) | 0.103 |
|  |  |  | Negative | 75(67.57) | 32(28.83) | 4(3.60) |  | 182(81.92) | 40(18.02) |  |
| rs591291 | C/T | anti-CCP | Positive | 197(36.62) | 242(44.98) | 99(18.40) | 0.775 | 636(59.11) | 440(40.89) | 0.546 |
|  |  |  | Negative | 29(34.94) | 36(43.37) | 18(21.69) |  | 94(56.63) | 72(43.37) |  |
|  |  | RF | Positive | 195(36.79) | 238(44.91) | 97(18.30) | 0.582 | 628(59.25) | 432(40.75) | 0.351 |
|  |  |  | Negative | 38(34.23) | 48(43.24) | 25(22.52) |  | 124(55.86) | 98(44.14) |  |
| rs11227209 | C/G | anti-CCP | Positive | 474(88.10) | 62(11.52) | 2(0.37) | 0.749 | 1010(93.87) | 66(6.13) | 0.505 |
|  |  |  | Negative | 75(90.36) | 8(9.64) | 0 |  | 158(95.18) | 8(4.82) |  |
|  |  | RF | Positive | 470(88.68) | 57(10.75) | 3(0.57) | 0.701 | 997(94.06) | 63(5.94) | 0.566 |
|  |  |  | Negative | 100(90.09) | 11(9.91) | 0 |  | 211(95.05) | 11(4.95) |  |
| rs35138901 | T/C | anti-CCP | Positive | 461(85.69) | 74(13.75) | 3(0.56) | 0.701 | 996(92.57) | 80(7.43) | 0.470 |
|  |  |  | Negative | 69(83.13) | 13(15.66) | 1(1.21) |  | 151(90.96) | 15(9.04) |  |
|  |  | RF | Positive | 456(86.04) | 71(13.40) | 3(0.56) | 0.536 | 983(92.74) | 77(7.26) | 0.263 |
|  |  |  | Negative | 91(81.98) | 19(17.12) | 1(0.90) |  | 201(90.54) | 21(9.46) |  |
| *ZFAS1* |  |  |  |  |  |  |  |  |  |  |
|  |  |  |  |  |  |  |  |  |  |  |
| rs237742 | C/T | anti-CCP | Positive | 202(37.55) | 272(50.56) | 64(11.89) | 0.088 | 676(62.83) | 400(37.17) | 0.217 |
|  |  |  | Negative | 30(36.14) | 36(43.37) | 17(20.48) |  | 96(57.83) | 70(42.17) |  |
|  |  | RF | Positive | 197(37.17) | 265(50.00) | 68(12.83) | 0.810 | 659(62.17) | 401(37.83) | 0.998 |
|  |  |  | Negative | 43(38.74) | 52(46.85) | 16(14.41) |  | 138(62.16) | 84(37.84) |  |
| rs73116127 | G/A | anti-CCP | Positive | 450(83.64) | 87(16.17) | 1(0.19) | 0.845 | 987(91.73) | 89(8.27) | 0.741 |
|  |  |  | Negative | 68(81.93) | 15(18.07) | 0 |  | 151(90.96) | 15(9.04) |  |
|  |  | RF | Positive | 442(83.40) | 88(16.60) | 0 | 0.090 | 972(91.70) | 88(8.30) | 0.574 |
|  |  |  | Negative | 91(81.98) | 19(17.12) | 1(0.90) |  | 201(90.54) | 21(9.46) |  |
| rs6125607 | C/T | anti-CCP | Positive | 249(46.28) | 229(42.57) | 60(11.15) | 0.950 | 727(67.57) | 349(32.43) | 0.981 |
|  |  |  | Negative | 39(46.99) | 34(40.96) | 10(12.05) |  | 112(67.47) | 54(32.53) |  |
|  |  | RF | Positive | 246(46.41) | 221(41.70) | 63(11.89) | 0.514 | 713(67.26) | 347(32.74) | 0.459 |
|  |  |  | Negative | 53(47.75) | 49(44.14) | 9(8.11) |  | 155(69.82) | 67(30.18) |  |
| rs6125608 | A/G | anti-CCP | Positive | 426(79.18) | 104(19.33) | 8(1.49) | 0.942 | 956(88.85) | 120(11.15) | 0.727 |
|  |  |  | Negative | 67(80.72) | 15(18.07) | 1(1.21) |  | 149(89.76) | 17(10.24) |  |
|  |  | RF | Positive | 427(80.57) | 96(18.11) | 7(1.32) | 0.200 | 950(89.62) | 110(10.38) | 0.081 |
|  |  |  | Negative | 81(72.97) | 28(25.23) | 2(1.80) |  | 190(85.59) | 32(14.41) |  |
